# How serving helps leading: mediators between servant leadership and affective commitment

**DOI:** 10.3389/fpsyg.2023.1170490

**Published:** 2023-07-03

**Authors:** Mayangzong Bai, Xinyi Zheng, Xu Huang, Tiantian Jing, Chenhao Yu, Sisi Li, Zhiruo Zhang

**Affiliations:** ^1^School of Public Health, Shanghai Jiao Tong University School of Medicine, Shanghai, China; ^2^Department of Pharmacy, Huashan Hospital, Fudan University, Shanghai, China; ^3^School of Business, Hong Kong Baptist University, Hong Kong, China

**Keywords:** servant leadership, affective commitment, job burnout, psychological safety, mediation model

## Abstract

**Introduction:**

Servant leadership has long been associated with maintaining employee’s affective commitment, yet the underlying mechanism remains unclear. Research from non-western cultures remains scarce.

**Methods:**

This study sought to fill in such research gap by introducing insights from social exchange theory perspective, and examined two potential mediators (viz., psychological safety and job burnout) with a largescale, representative Chinese sample.

**Results:**

A total of 931 staffs in a Chinese hospital were surveyed, and structural equation models revealed that psychological safety (indirect effect = 0.052, 95% Bootstrap CI = [0.002, 0.101]) and job burnout (indirect effect = 0.277, 95% Bootstrap CI = [0.226, 0.331]) parallelly (and partially) mediated the effect of servant leadership on affective commitment. Moreover, these effects held the same between permanent and temporary staffs, as well as between male and female staffs.

**Discussion:**

Results suggested that a leader’s orientation to care, validate, and respond to their followers’ needs was effective in creating a psychological safe environment and downplaying job burnout in workplace, in exchange to which, followers remained affectively committed to their organization in a long term. Not only did this study contribute to existing literature by providing non-western data for service leadership research, it also provided a deeper understanding of associated mechanisms of how servant leadership might cast on talent retain and organizational development in a long term. These mechanisms shed light on how serving helps leading and advocate servant leadership for hospitals, as well as other serving organizations.

## Introduction

Human resources are the most important factor in various organizations (Specchia et al., 2021), despite the pervasive existence of many management dilemmas, e.g., high employee turnover, low workplace engagement, and lack of organizational ownership especially in recent decade (Gile et al., 2022; Stanimirović and Pribaković, 2022; Ekingen et al., 2023). One crucial factor to prevent these dilemmas is the maintenance of employees’ affective commitment, which was found negatively related to both turnover intentions and actual turnover behaviors, and positively related to job performance and willingness to stay in the current organization (Fleig-Palmer and Rathert, 2015; Deschênes, 2023; Lado et al., 2023). As affective commitment is associated with various favorable organizational and personal outcomes [e.g., job performance, organizational citizenship behaviors, absenteeism, and turnover (Heffner and Rentsch, 2001)], only by retaining talents can organizations remain core competencies, and promote faster and better growth (Mercurio, 2015).

Previous research suggested that servant leadership was a strong positive predictor of affective commitment (Fernandez and Pitts, 2007; Higgs and Rowland, 2010; Van der Voet et al., 2016). Yet these findings were somewhat limited in its lack of non-western research samples and its lack of underlying mechanisms (Avolio et al., 2009; Higgs and Rowland, 2010). In response to which, this study examined the association of servant leadership and affective commitment in a Chinese public, comprehensive hospital, and further examined two potential mediators (*viz.*, psychological safety and job burnout) from a social exchange theory perspective.

### Theoretical background and hypotheses

Affective commitment is the core of organizational commitments (Mercurio, 2015; Noraazian and Khalip, 2016), it refers to employees’ (emotional) dependence on the organization, and the extent to which employees have the same values and objectives as the organization leads (Haider et al., 2019). Employees high in affective commitment believe in and accept the organization’s goals and values, intend to stay with the organization in a long-term, and commit to providing quality service on behalf of the organization (Shao et al., 2022).

Previous research reveals that servant leadership plays an important role in retaining employees’ affective commitment among various challenges (He et al., 2008; Mercurio, 2015; Goestjahjanti et al., 2022). More importantly, social exchange theory (SET) proposes that social exchange processes involve a series of interdependent and contingent interactions that evokes workplace obligation (Blau, 1974; Richard and Emerson, 1976), for which leadership plays an important role (Liden et al., 1997).

### From servant leadership to affective commitment

Servant leaders care for their followers’ personal needs and interests, and focus on the benefits of organizations and communities rather than their own interests (Eva et al., 2019). Compared to others (e.g., transformational leadership), this type of leadership is more likely to create social exchange relationships between leaders and their followers. According to SET, it is expected that when servant leaders provided employees with good working atmospheres, opportunities to improve professional skills, emotional support in need, in exchange, employees might develop a sense of identity and belonging to the organization (Cropanzano and Mitchell, 2005; Dahleez et al., 2021). Hence, it was hypothesized that *servant leadership positively affected affective commitment* (H1). In addition, this positive association could be mediated by (increasing) psychological safety and/or (decreasing) job burnout in workplace. Rationales were articulated below.

### Psychological safety: willingness to exchange

Psychological safety refers to the belief that workplace is safe for interpersonal risk-taking such that presenting of self-image, career, or status is free from the fear of adverse effects (Edmondson, 1999; Frazier et al., 2017). When employees have a high level of psychological safety, they feel free to speak their minds, actively exchange work-related knowledge and information, and are energized, creative, and effective in performance even in rapidly changing environments such as healthcare revolution (Edmondson and Lei, 2014).

Servant leadership has a positive effect on the attitudes and behaviors of employees, with leaders putting employees’ interests first, helping them develop and grow, and building good relationships with their followers. The recognition of being cared for, respected, and helped by their leaders might also grant the psychological safety to take challenges, seize opportunities, even taking risks to introduce different ideas; should there be any concern for adverse consequences related, employees are less fearful as they trust their servant leaders to “get their back” (Yan and Xiao, 2016). Therefore, it was presumed that servant leadership could increase employees’ psychological safety. According to SET, exchange relationships are characterized by the mutual caring of both parties’ interests (*cf.* Clark). With psychological safety granted, employees would be more willingly in taking responsibilities, stepping-out of comfort zones, and even bringing-about beneficial changes in the organization (Edmondson and Lei, 2014). In other words, employees who felt high in psychological safety in workplace would trust, identify, and dedicate more to the organization, resulting in both personal and organizational growth that could reinforce their affective bonding to the organization (Chandrasekaran and Mishra, 2012; Frazier et al., 2017; Li et al., 2022). Therefore, it was hypothesized that *servant leadership could boost affective commitment by enhancing the experience of psychological safety* (H2).

### Job burnout: resources to exchange

Job burnout is a progressive psychological response to chronic job-related stress and includes three components: emotional exhaustion, which refers to the excessive depletion of one’s emotional resources, resulting in feelings of emotional and physical burden and strain; depersonalization, which refers to a decreased ability to empathize with clients, and a cold, overly distant attitude; and a decreased sense of personal accomplishment, which refers to a loss of job satisfaction and competency (Lombardero-Posada et al., 2022; Yıldırım et al., 2023). Healthcare providers are exposed to many stressors including but not limited to challenges of clinical work, competing demands, conflicting roles, and relationships with leadership (Demin et al., 2017), which resulted in high prevalence of job burnout in this industry (Hall et al., 2016; Chirico et al., 2023). Especially with the shortage of human resources after the global Covid-19 pandemic, occupational hardship involving burnout, anxiety, and lack of career-related psychological security had become more and more prevalent(Chirico et al., 2022).

Studies have shown that servant leadership is negatively associated with job burnout, especially among medical staffs (Cai et al., 2013; Harju et al., 2018; Ma et al., 2021). Servant leadership is very likely to minimize the chances of having emotional exhaustion, depersonalization, or lack of personal accomplishment, because leaders of this type of care not only the performance but also the well-being of their followers’. More importantly, employees with less job burnout have more resources to reward the organization, such as passionate, devoted, and skilled workplace performance. In such healthy social exchange processes, chances are high that employees would like to serve the organization in long-term, as working here becomes rewarding and enjoyable (Blau, 1974; Richard and Emerson, 1976). In other words, employees with servant leaders are less likely to have their organizational commitment jeopardized by the violation of their identification to the organization (Spence Laschinger et al., 2009). Therefore, it was hypothesized that *servant leadership could boost affective commitment by reducing the experience of job burnout* (H3).

Finally, job burnout could be a function of age or gender given employees at different life stages or in different family roles could face various life work conflicts, psychological safety could be a function of profession or career stage due to different organizational roles (Artz et al., 2022). Possibilities that the proposed mediation effects could be conditioned by demographic and/or professional characteristics could not be rule out in advance. Therefore, this study intended to explore whether the proposed model differed at demographic and/or professional features.

### The present study

The objectives of the current study were to (a) examine the proposed parallel mediation effects of psychological safety and job burnout on the relationship from servant leadership to employee’s affective commitment; and (b) to explore the potential differences of the above associations among different demographic and professional characteristics. In short, this study served as a possible extension of the social exchange theory, such that it examined whether servant leadership could evoke beneficial exchange processes via increasing psychological safety (the willingness to exchange) and decreasing job burnout (the resources to exchange).

## Methods

### Participants and procedure

A convenience sampling was conducted at a local comprehensive, second-grade hospital in Shanghai, China November 2021. This hospital consisted of various clinical departments^1^ it was typically representative of the general hospitals of its level in China. All staffs (*n* = 1925) were invited to participate, and a total of 931 valid responses (*M*_age_ = 35.58 years, *SD* = 9.26; 80.42% were female) were collected. All participants provided informed consent.

This Research was approved by the Research Ethics Committee of Shanghai Jiao Tong University School of Medicine (reference ID: SJTUPN-202202). Hospital department heads were contacted in advance to introduce the purpose and procedure of this study. They were also instructed to encourage employees to participate in this anonymous, online survey distributed via a mature Chinese online survey platform (Wenjuanxing, Changsha, China). Multiple procedures were adopted to prevent common method bias frequently observed in self-report surveys, including, (a) the randomization of question orders, and (b) the insertion of reverse coded questions. In addition, the response window was specified as 3 days after the release of the survey link, all respondents were encouraged to response in a one-time and in privacy. Hospital staffs were further required to not to discuss the study or their own responses with each other until the study ended.

### Measures

Well established psychological scales were adopted to measure servant leadership, psychological safety, job burnout, and affective commitment. The original English versions were translated into Chinese following standard procedures of translation and back-translation. Wording was modified to fit the Chinese healthcare environment, when necessary. All translated scales were tested for content and construct validity, and three items were removed for low factor loadings and cross-loadings on multiple dimensions (see Supplementary Table 1 for details). For each construct, a composite score was created by averaging all items such that higher scores indicate higher levels of that construct.

#### Servant leadership

Twenty-seven items were adapted from Liden et al.’s (2008) servant leadership scale (e.g., “My manager does what she/he can do to make my job easier.”; 1 = *strongly disagree* to 5 = *strongly agree*) (Liden et al., 2008) with one original item excluded.

#### Psychological safety

Seven items were adapted from Edmondson’s (1999) psychological safety scale (e.g., “If you make a mistake in your department, it is often held against you.”; 1 = *very strongly disagree* to 7 = *very strongly agree*) (Edmondson, 1999).

#### Job burnout

Twenty items were adapted from the Maslach Burnout Inventory–Human Service Survey (MBI-HSS; e.g., “I feel depressed at work.”; 1 = *never* to 7 = *every day*) (Maslach, 1996) with two original items excluded.

#### Affective commitment

Six items were adapted from Meyer et al.’s (1993) affective commitment scale (e.g., “I would be very happy to spend the rest of my career with this hospital.”; 1 = *very strongly disagree* to 7 = *very strongly agree*) (Meyer et al., 1993).

Finally, participants were also asked to report their age, gender, marital status, education level, occupation, and annual income.

### Analytical scheme

All analyses were conducted in SPSS 26.0, Jamovi 2.3.21, and SPSS AMOS 26.0. Psychometric properties of the translated scales were examined before hypothesis testing. Reliability was examined via internal consistency, and a McDonald’s *ω* > 0.700 was considered acceptable (Trizano-Hermosilla and Alvarado, 2016). Validity was examined via confirmatory factor analysis (CFA). Scales were individually analyzed according to theoretical structures, and model fits and factor loadings were evaluated in conjunction to assess structural validities against pre-determined criteria (namely, goodness-of-fit index (GFI), comparative fit index (CFI), and Tucker-Lewis index (TLI) > 0.900; *χ*^2^/*df* < 3, and root mean square error of approximation (RMSEA) < 0.080) (Lt and Bentler, 1996; Schermelleh-Engel et al., 2003). In addition, scales were modeled together upon their own factor models to examine the convergent and discriminate validities via average variance extracted (Fornell and Larcker, 1981) [AVE; which was expected to be larger than the correlation coefficients among the corresponding factors and composite reliability (CR > 0.700) (Peterson and Kim, 2013)]. Harman’s single-factor test was used to probe common method bias (Podsakoff et al., 2003; Tehseen et al., 2017).

For hypothesis testing, the proposed parallel mediation effects were examined using SPSS PROCESS macros 22.0 (Model 4; bootstrap samples = 5,000) (Hayes, 2017). Variance inflation factor (VIF) was adopted to evaluate multicollinearity, and given the cross-sectional design, a relatively strict cut-off value was determined prior to data analysis such that a VIF smaller than 2.5 was considered acceptable.

## Results

**Figure 1:**
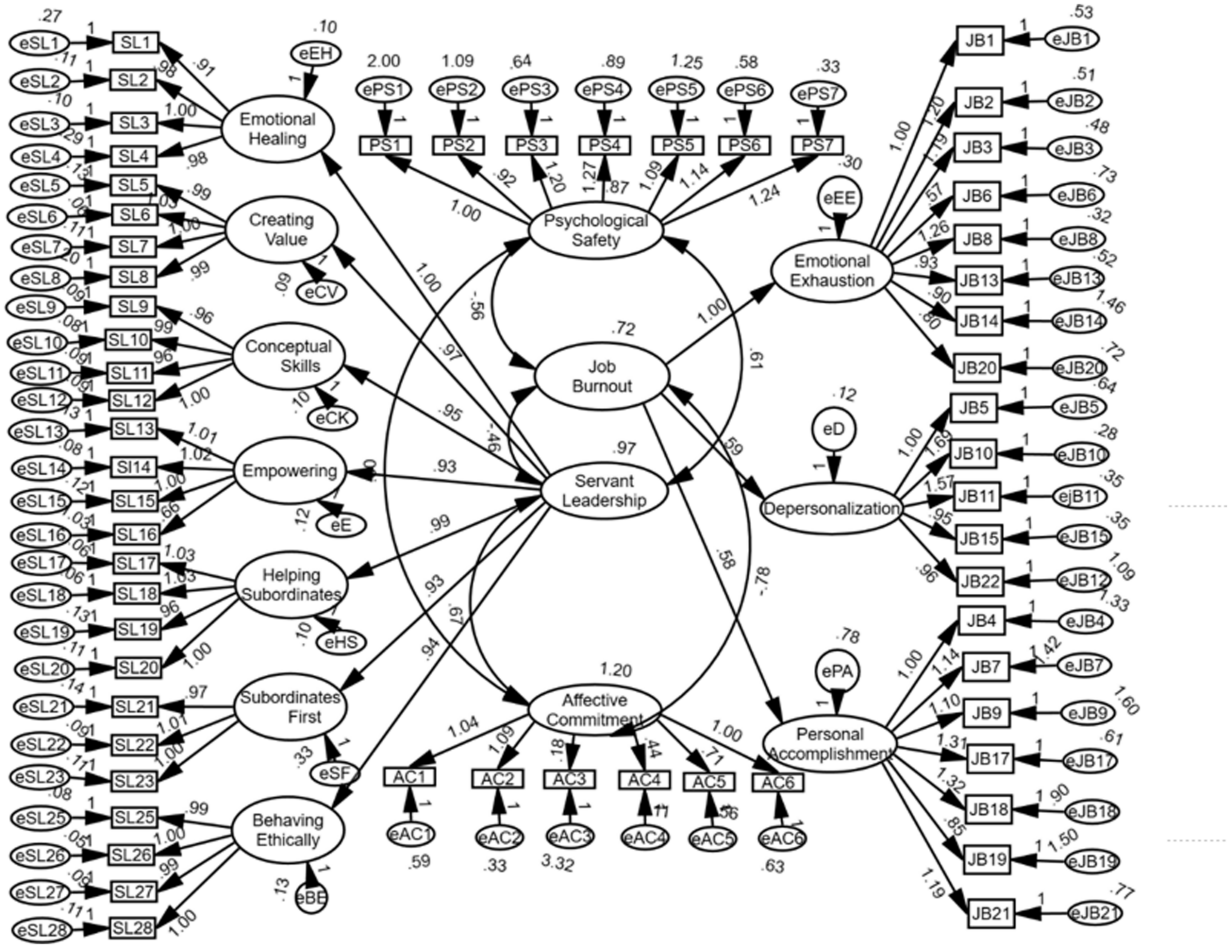
Discriminate validity test result. SL, servant leadership; PS, psychological safety; JB, job burnout; AC, affective commitment.

Sample characteristics were reported in Table 1.^2^ Descriptive statistics, McDonald’s *ω*s, and bivariate correlations of key variables are presented in Table 2. In psychometric tests, one item from servant leadership scale was excluded due to unsatisfactory factor loading and two were excluded from job burnout scale due to cross loading (see Supplementary Table 1 for details). After revision, all translated scales exhibited satisfactory reliability (McDonald’s *ω*s ≥ 0.792), and well-supported structural and discriminate validities (model fit: *χ*^2^*/df* = 3.924, CFI = 0.923, TLI = 0.919, RMSEA = 0.057, SRMR = 0.064; see Figure 1; Table 2). The first factor revealed by the Harman’s single-factor test did not capture most of the variance (45.95%), suggesting no substantial common method bias.

**Table 1:** Sociodemographic characteristics and affective commitment distribution across these characteristics.

Demographic characteristics	*n* (%)	*M*	*SD*	*F*/*t*	*p*
*Gender*					
Female	758 (80.418)	4.731	1.064	0.333	0.564
Male	173 (18.582)	4.940	1.088		
*Age group*					
<30	292 (31.364)	4.854	1.091	2.305	0.075
31 ~ 40	361 (38.776)	4.859	1.142		
41 ~ 50	210 (22.556)	4.943	1.007		
>50	60 (6.445)	5.231	0.987		
*Marital status*					
Single	264 (28.357)	4.805	1.137	1.683	0.195
Married	667 (71.644)	4.939	1.063		
*Education*					
High-school or below	271 (29.108)	4.935	1.143	0.385	0.681
Bachelor’s degree	522 (56.068)	4.900	1.072		
Postgraduate	138 (14.823)	4.836	1.022		
*Profession*					
Doctor	217 (23.308)	4.835 ^a, b^	1.015	9.754	< 0.001
Nurse	567 (60.902)	5.009 ^a^	1.079		
Medical technicians	147 (15.789)	4.582 ^b^	1.148		
*Job title*					
Senior	68 (7.304)	5.240 ^a^	0.931	3.547	0.014
Intermediate	363 (38.990)	4.882 ^a^	1.094		
Junior	452 (48.550)	4.840 ^a, b^	1.081		
Other	48 (5.156)	5.142 ^a^	1.181		
*Type of contract*					
Permanent	664 (71.321)	4.913	1.075	0.990	0.320
Temporary	267 (28.679)	4.871	1.112		
*Annual income*					
100,000 or below	244 (26.208)	4.863	1.115	1.230	0.298
100–200,000	598 (64.232)	4.908	1.086		
200,000–300,000	81 (8.700)	4.895	0.994		
300,000 or above	8 (0.859)	5.604	0.926		
*Shift type*					
Dayshift	341(36.627)	5.122 ^a^	1.043	11.395	< 0.001
Two shifts	165 (17.723)	4.757 ^b^	1.093		
Three shifts	425 (45.650)	4.780 ^b^	1.090		

Annual income was in Chinese Yuan. Group means with different superscriptions were significantly different from each other.

**Table 2:** Descriptive statistics, reliabilities, and bi-variate correlations between variables of interest.

	*M*	*SD*	McDonald’s *ω*	1	2	3	4	Composite reliability
1 Servant leadership	3.683	0.942	0.990	(0.636)				0.988
2 Psychological safety	5.039	1.116	0.896	0.604	(0.616)			0.918
3 Job burnout	2.920	0.819	0.918	−0.509	−0.554	(0.461)		0.962
4 Affective commitment	4.901	1.086	0.792	0.467	0.443	−0.605	(0.727)	0.941

All correlations were significant at *p* < 0.001. Values on the diagonal represent AVE.

### Mediating effects of psychological safety and job burnout

**Figure 2:**
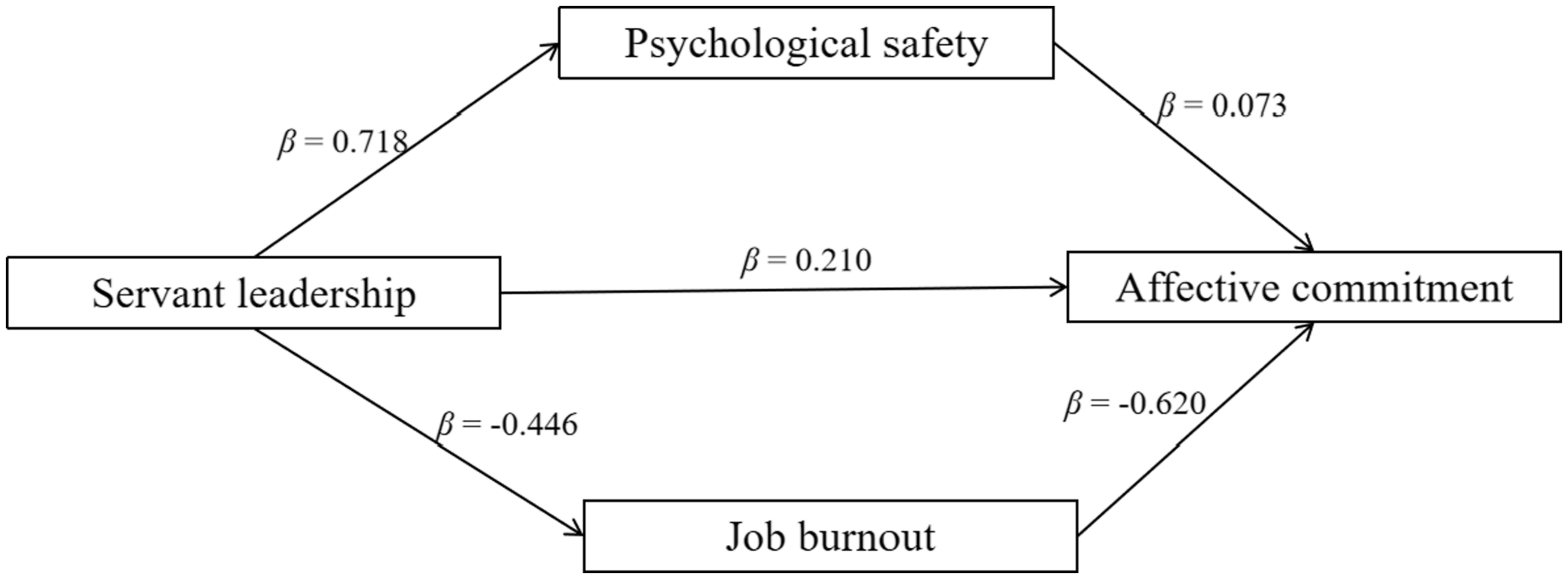
Results for the mediation analysis. Standardized coefficients were reported. All coefficients were significant at *p* < 0.001.

Mediation analysis was performed to examine whether staff members’ psychological safety and job burnout (in parallel) mediated the effect of employees’ perceived servant leadership on their affective commitment, with shift type and income level as control variables (Table 3). VIFs were below 2.00 for all independent variables, suggesting no substantial multicollinearity. Servant leadership was positively associated with affective commitment while psychological safety and job burnout simultaneously and partially mediated the above association. Specifically, after accounting for the indirect effects via psychological safety and job burnout, the direct effect remained significant. Together, this mediation effect accounted for 61.055% of the total effect from servant leadership to affective commitment. A further contrast of the two indirect effects suggested that the mediation effect via job burnout was stronger than the effect via psychological safety (Figure 2).

**Table 3:** Results of the mediation model.

	Whole sample				Permanent staffs				Temporary staffs			
	*B*	*SE*	LLCI	ULCI	*B*	*SE*	LLCI	ULCI	*B*	*SE*	LLCI	ULCI
Total effect (SL → AC)	0.538	0.034	0.471	0.605	0.496	0.039	0.420	0.573	0.681	0.071	0.541	0.820
Direct effect (SL → AC)	0.210	0.039	0.134	0.285	0.227	0.045	0.138	0.315	0.170	0.078	0.016	0.323
Total indirect effect	0.327	0.031	0.271	0.393	0.270	0.035	0.204	0.340	0.511	0.076	0.375	0.668
Path 1:SL → PS → AC	0.052	0.025	0.002	0.101	0.033	0.029	−0.025	0.091	0.113	0.053	0.013	0.220
Path 2:SL → JB → AC	0.277	0.027	0.226	0.331	0.237	0.028	0.184	0.294	0.398	0.070	0.273	0.544
Contrast of paths 1 vs. 2	−0.224	0.042	−0.309	−0.144	−0.204	0.046	−0.295	−0.116	−0.285	0.098	−0.486	−0.103

*n* = 908. SL, servant leadership; PS, psychological safety; JB, job burnout; AC, affective commitment. LLCI and ULCI stand for lower and higher limit of the 95% bootstrapping confidence interval, respectively.

### Multi-group analyses

**Figure 3:**
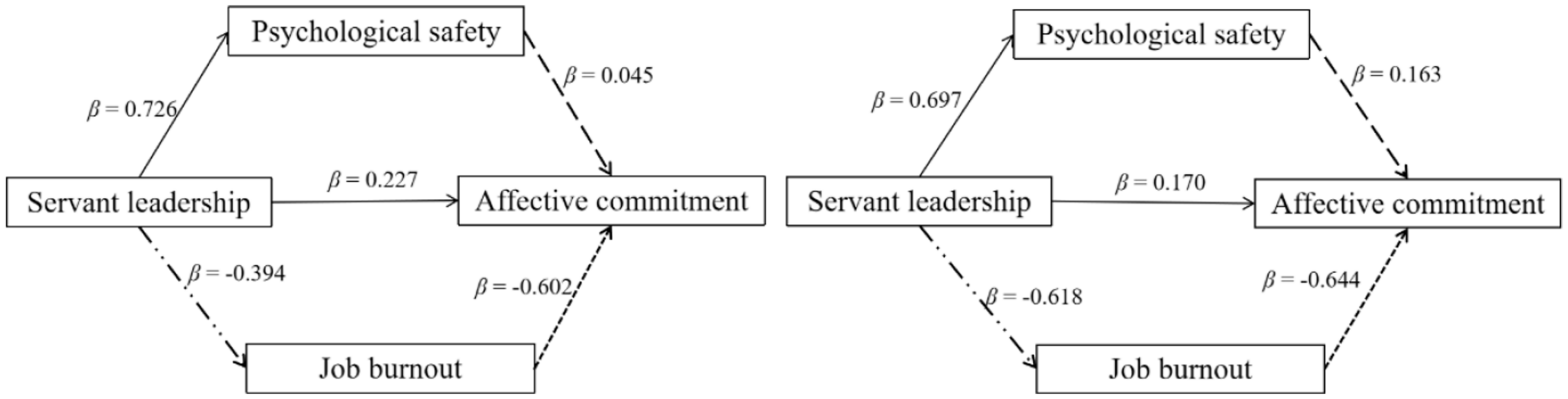
Comparing the mediation effects between permanent (left panel) versus temporary (right panel) staffs. Standardized coefficients were reported. All coefficients were significant at *p* < 0.001. Solid lines indicate no statistical difference between the corresponding effects for permanent and temporary staffs.

Multi-group analyses were conducted to examine whether the above mediation model held equivalently for (a) male versus female participants, (b) permanent staff versus temporary staff participants.^3^ Critical ratios for parameter differences were adopted for path comparisons. Results revealed that the negative effect of servant leadership on job burnout, the negative effect of job burnout on affective commitment, and the positive effect of psychological safety on affective commitment were greater in terms of the magnitudes for temporary staffs than for permanent staffs (Figure 3). However, the two mediation effects were not significantly different between the two groups (*B*s < −0.002, *p* > 0.071). And accordingly, the total indirect effect was not significantly different (*B* = −0.162, *p* = 0.132, 95% CI = [−0.385, 0.047]) between the two groups, either. No gender difference was found across all pairs of paths (see Supplementary Table 2 for details).

## Discussion

The results of this study revealed that, as predicted, psychological safety and job burnout parallelly mediated the relationship between servant leadership and affective commitment such that servant leadership could enhance affective commitment by increasing employees’ psychological safety and reducing job burnout. These mediation effects held the same between permanent and temporary staffs, and between male and female staffs.

Results supported the proposed mediators (*viz.*, psychological safety and job burnout), suggesting that servant leadership could promote employees’ affective commitment via not only creating a psychological safe environment (Edmondson and Lei, 2014; Azam et al., 2017; Frazier et al., 2017; Li et al., 2022) but also granting resources to recover from work stress. Furthermore, it is noteworthy that indirect effect via job burnout was somewhat stronger than that via psychological safety. One possible reason was that healthy leaderships could help employees practice self-regulation and develop coping flexibility especially when job resources were drained (Bakker and de Vries, 2021), which could be applied to a variety of different scenarios, including those where voice-ups were encouraged. Therefore, job burnout outperformed psychological safety as the mediator here.

Although overall mediation effects held the same between temporary and permanent staffs, path analyses revealed that servant leadership decreased job burnout to a larger extent for the former than for the latter. This subtle difference suggested a strategic provision of servant leadership-oriented management toward temporary staffs, for that these staffs tended to be less stable and less resourceful in workplace and therefore more vulnerable to job burnout, yet as results revealed, these staffs tended to benefit more from caring leaders, compared to their permanent counterparts.

### Theoretical implications

Previous research on servant leadership mainly focused on the antecedences and consequences, little research attention was directed to the mechanisms why and how serving helps leading. As researchers pointed out, this field would benefit from in-depth examination of the mechanism(s) associated with servant leadership (Eva et al., 2019). In responding to which, this study examined and revealed that serving others’ needs brought benefits via not only organizational psychological climate but also followers’ affective well-being. Additionally, this study contributed to existing literature by providing non-western data for the examination of servant leadership.

A distinguishable feature of this study was that it examined behavioral tendencies (servant leadership and affective commitment) instead of concrete behaviors in social exchange processes (Cook et al., 2013). From leaders’ end, a servant orientation provides an umbrella test of resources such as love (i.e., an expression of affectionate regard, warmth, or comfort), status (i.e., an evaluative judgment that conveys prestige, regard, or esteem), and information (i.e., advice, opinions, instruction, or enlightenment) among the six types of critical resources proposed by the resource theory of social exchange (Tolin et al., 2003). From subordinates’ end, affective commitment is one of the socioemotional outcomes that address’s one’s social and esteem needs and conveys a sense of being valued and/or treated with dignity (Shore et al., 2001; Cook et al., 2013). While concrete behaviors (e.g., short-term monetary reward or cooperation) might be more convenient to measure, behavioral tendencies granted more generalizability in research findings. More importantly, the theoretical model tested here shed more light on those long-term, socioemotional outcomes rather than short-term, tangible ones, which were more important for the organizational development (Cook et al., 2013). Results added new empirical evidence of social exchange theory in leadership research. Using psychological safety and job burnout as proxies, results also highlighted that servant leadership contributed to beneficial social exchange processes via the securing of both willingness and resources from employee’s perspective.

### Practical implications

Based on the results of this study, some practical implications are suggested. First, psychological safety and burnout significantly mediated the relationship between servant leadership and affective commitment, suggesting that managers need to appreciate how their servant leadership behaviors affect employees’ affective commitment. It is essential to clearly understand that servant leadership is an important part of employees’ increased affective commitment. The more followers perceive servant behavior, the more likely they are to commit to staying with the organization. In addition, this study shows that influencing affective commitment through burnout is stronger than psychological safety, suggesting that managers should prioritize reducing employee burnout. Finally, the mediation model holds across groups, suggesting that employees of different genders and employment forms can increase their affective commitment through servant leadership in the hospital context.

### Study limitation and future direction

Several limitations need consideration. First, the cross-sectional design limited the examination of the causal relationships among the investigated variables, longitudinal studies are recommended to further the understanding of the mechanisms revealed here. Second, this study focused on the effects of servant leadership behaviors on individual-level mediators, while team-level mediators might play important roles as well, e.g., collaborative team atmosphere inspired by servant leadership’s other-interests orientation. Future studies might consider integrating mechanisms at both individual- and team- levels. Thirdly, authors encouraged some replication of the current findings in hospitals with different specialties and scales, as well as in other serving organizations to examine the generalizability of the theoretical model. Fourthly, though psychological safety and job burnout could proximate well the willingness and resources to exchange, authors recommended future research to adopt other variables (e.g., citizenship behaviors) for conceptual replication. Finally, this study focused on the perceived servant leadership, future studies could further examine actual levels (e.g., leaders’ self-report) to provide a comprehensive picture as well.

## Conclusion

This study revealed that servant leadership could promote hospital employees’ affective commitment by (increasing) psychological safety and (decreasing) job burnout, and job burnout outperformed psychological safety as the mediator. Our findings make significant contributions to the field of servant leadership and shed light on several new research directions.

## Data availability statement

Data would be available upon request made to corresponding authors.

## Ethics statement

The studies involving human participants were reviewed and approved by the Research Ethics Committee of Shanghai Jiao Tong University School of Medicine (reference ID: SJTUPN-202202). The patients/participants provided their written informed consent to participate in this study.

## Author contributions

MB, SL, ZZ, and XZ contributed to conception and design of the study, and organized the database. MB, XH, TJ, and CY performed the statistical analysis. MB wrote the first draft of the manuscript. SL, XZ, and XH wrote sections of the manuscript. All authors contributed to manuscript revision, read, and approved the submitted version.

## Conflict of interest

The authors declare that the research was conducted in the absence of any commercial or financial relationships that could be construed as a potential conflict of interest.

## Publisher’s note

All claims expressed in this article are solely those of the authors and do not necessarily represent those of their affiliated organizations, or those of the publisher, the editors and the reviewers. Any product that may be evaluated in this article, or claim that may be made by its manufacturer, is not guaranteed or endorsed by the publisher.

## Footnotes

^1^This hospital consisted of 14 clinical departments (e.g., surgery, internal medicine, gynecology, and pediatrics), 4 medical technology departments (*viz.*, multidisciplinary laboratories, medical imaging services, pharmacology/pharmacy), 15 administrative departments (e.g., medical affairs, hospital infection management), and over 1,000 hospital beds.

^2^Data would be available upon requests toward the correspondence authors.

^3^Permanent staff group (*n* = 664) and temporary staff group (*n* = 267).
