# Supplementary material for: How serving helps leading: mediators between servant leadership and affective commitment
Source: Front Psychol. 2023 Jul 3;14:1170490. doi: 10.3389/fpsyg.2023.1170490 (PMC10351042; doi:10.3389/fpsyg.2023.1170490)
Supplement: Supplementary file 1 [file Data_Sheet_1.PDF]

Supplementary Table 1
Structural validity test results and revision notes.

	χ^2/df	p	CFI	TLI	RMSEA	SRMR	Revision notes
Servant leadership [a]	5.215		0.970	0.966	0.068	0.029	One original item was excluded due to unsatisfactory factor loading.
Psychological safety	4.299		0.993	0.980	0.060	0.017	No item was removed.
Job burnout	6.061		0.933	0.917	0.075	0.058	Two original items were excluded due to cross-loading.
Affective commitment	1.702		0.998	0.996	0.028	0.014	No item was removed.

Note. [a] First-order structure was adopted. These structural validity tests were conducted such that each individual scale was modeled according to its theoretical structure, results in general suggested that all individual structural validity was acceptable after revision.

Supplementary Table 2.
Multi-group analyses results.

	**Male**				**Female**			
	Estimate	*p*	*SE*	95% CI	Estimate	*p*	*SE*	95% CI
SL → PS	0.581	< .001	0.069	[0.398, 0.736]	0.675	< .001	0.045	[0.595, 0.746]
SL → JB	-0.428	< .001	0.053	[-0.565,-0.261]	-0.519	< .001	0.032	[-0.591,-0.434]
SL → AC	0.075	.279	0.068	[-0.079, 0.242]	0.131	< .001	0.044	[0.054, 0.216]
PS → AC	0.544	< .001	0.077	[0.323, 0.727]	0.421	< .001	0.051	[0.315, 0.526]
JB → AC	-0.472	< .001	0.092	[-0.647,-0.311]	-0.474	< .001	0.054	[-0.565,-0.390]
	**Regular**				**Contract-based**			
	Estimate	*p*	*SE*	95% CI	Estimate	*p*	*SE*	95% CI
SL → PS	0.626	< .001	0.061	[0.487, 0.739]	0.663	< .001	0.046	[0.570, 0.736]
SL → JB	-0.567	< .001	0.056	[-0.675,-0.424]	-0.478	< .001	0.03	[-0.554,-0.387]
SL → AC	0.103	.084	0.069	[-0.017, 0.223]	0.146	< .001	0.044	[0.065, 0.234]
PS → AC	0.581	< .001	0.074	[0.421, 0.711]	0.388	< .001	0.05	[0.271, 0.497]
JB → AC	-0.384	< .001	0.064	[-0.530,-0.258]	-0.503	< .001	0.062	[-0.596,-0.410]

Note. Gender difference, $p = .156$; and employment difference, $p < .001$. Abbreviations: SL = Servant leadership; PS = Psychological safety; JB = Job burnout; and AC = Affective commitment.

Supplementary Table 3.
Differences between path parameters in multiple-group analyses.

Critical ratios (Measurement weights)	SL → PS	PS → AC	SL → AC	SL → JB	JB → AC
Gender: Female vs. male					
	1.498				
	-0.496	-0.976			
	-4.831	-4.863	0.905		
	-12.244	-11.803	-6.379	-2.106	
	-13.877	-13.055	-8.601	-4.897	0.145
Employment: Regular versus contract-based					
	-0.226				
	0.658	3.399			
	-6.222	-3.692	-0.411		
	-15.790	-13.021	-9.721	-3.618	
	-14.223	-11.202	-8.235	-2.046	3.704

Note. Abbreviations: SL = Servant leadership; PS = Psychological safety; JB = Job burnout; and AC = Affective commitment.
